# A Comprehensive Review of Wetting Transition Mechanism on the Surfaces of Microstructures from Theory and Testing Methods

**DOI:** 10.3390/ma15144747

**Published:** 2022-07-06

**Authors:** Xiao Wang, Cheng Fu, Chunlai Zhang, Zhengyao Qiu, Bo Wang

**Affiliations:** 1 Key Laboratory of Advanced Functional Materials, Education Ministry of China, Faculty of Materials and Manufacturing, Beijing University of Technology, Beijing 100124, China; b202009004@emails.bjut.edu.cn (X.W.); zclyouxiang@emails.bjut.edu.cn (C.Z.); qiuzhengyao@emails.bjut.edu.cn (Z.Q.); 2 China Classification Society Quality Assurance Ltd., Beijing 100006, China; sishiergeren@126.com

**Keywords:** wetting transition, superhydrophobic, microstructures, contact angle

## Abstract

Superhydrophobic surfaces have been widely employed in both fundamental research and industrial applications because of their self-cleaning, waterproof, and low-adhesion qualities. Maintaining the stability of the superhydrophobic state and avoiding water infiltration into the microstructure are the basis for realizing these characteristics, while the size, shape, and distribution of the heterogeneous microstructures affect both the static contact angle and the wetting transition mechanism. Here, we review various classical models of wettability, as well as the advanced models for the corrected static contact angle for heterogeneous surfaces, including the general roughness description, fractal theory description, re-entrant geometry description, and contact line description. Subsequently, we emphasize various wetting transition mechanisms on heterogeneous surfaces. The advanced testing strategies to investigate the wetting transition behavior will also be analyzed. In the end, future research priorities on the wetting transition mechanisms of heterogeneous surfaces are highlighted.

## 1. Introduction

Surface wettability is one of the most vital properties of a solid surface. The wettability of a solid surface is determined by the chemical properties and the micro-texture of the surface [1,2,3,4]. Young’s equation has been used to describe wetting on a smooth surface from 1805 [5]. However, real surfaces are seldom perfectly smooth. Hence, the Wenzel (W) and Cassie–Baxter (C–B) states are the two main kinds of solid–liquid wetting states on the micro-structured surfaces [6,7]. The description of the W state is based on the hypothesis that the water droplet completely penetrates the grooves of a rough surface, while the C–B state assumes the water droplet is suspended on the top of the micro-structured surface, which results in a composite interface. Compared with the W state, the C–B presents the high apparent contact angle (CA) and the low contact angle hysteresis. Maintaining the stability of the C–B state and avoiding the intrusion of water into the microstructure are essential preconditions for realizing self-cleaning, water-repelling, and anti-sticking properties [8].

The C–B state is not always stable, and the transition from the Cassie–Baxter to the Wenzel state (C–B/W) can occur when it is induced by various factors, such as pressurization, [9] vibrations, [10], and the gravity of the droplet itself [11,12]. Therefore, exploring the conditions of C–B state stability and understanding the C–B/W transition mechanisms have been a central topic in the study of superhydrophobic surfaces. Over 900 journal papers studying wetting transition mechanisms have been published that cover materials science, engineering, physics, and chemistry science technology.

Here, we summarize the recent advances in the theoretical study and testing methods of the wetting state transition mechanism. In the next section, the theory of fundamental wetting models is discussed, which is followed by the description of the static contact angle model. The advanced wettability transition mechanism is presented in Section 4, and a comprehensive overview of the wetting stability testing methods is provided in Section 5. To conclude, a brief outlook for future research directions is proposed.

## 2. Fundamental Wetting Theory

The first investigation of wetting phenomena can be traced back to 1612, which was presented by Galileo through his report, “Bodies That Stay atop Water, or Move in it” [13]. Over the recent few decades, great progress in the wetting theories has been developed to describe the wetting state models. In this section, the fundamental wetting theories are summarized.

**Figure 1:**
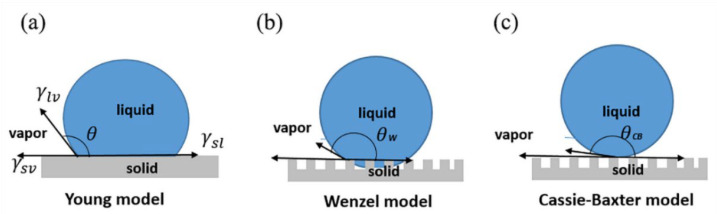
Various states of droplet on a solid surface. (**a**) Young model, (**b**) Wenzel model, and (**c**) Cassie–Baxter model.

In 1805, Thomas Young proposed the primary law of wetting with a water droplet on a flat and smooth surface, as shown in Figure 1a [5]. Young’s equation is given as:

(1) $$\cos\theta =\frac{\gamma_{sv}-\gamma_{sl}}{\gamma_{lv}}$$

where θ is the static contact angle, $\gamma_{sv}$,$\;\gamma_{sl}$, and $\gamma_{lv}$ are the solid–vapor, solid–liquid, and liquid–vapor surface tensions, respectively. Based on the value of *θ*, the property of the surface can be divided into the hydrophobic (*θ* > 90°) and hydrophilic (*θ* < 90°) surfaces [14].

Since Young’s equation is valid only for smooth and homogenous surfaces, in 1936, Wenzel modified Young’s model and introduced the roughness factor (*r*) to describe the wettability phenomena of the micro-structured surfaces, as shown in Figure 1b [3]:

(2) $$\cos\theta_{W}=r\frac{\gamma_{sv}-\gamma_{sl}}{\gamma_{lv}}=r\cos\theta$$

where $\theta_{W}$ is the static contact angle under the Wenzel state. The roughness factor (*r*) is defined as the ratio of the true surface area and planar surface, which is higher than 1 for a microstructured surface. In 1945, Cassie–Baxter described another wetting state for the droplet on microstructured surface, shown in Figure 1c. The model supposed the droplet is suspended on the top of the micro-structured surface, which results in a composite interface [4]. In the C–B model, the apparent contact angle is influenced by the contribution of two different phases, as described in the equation below:

(3) $$\cos\theta_{CB}=f_{sl}\cos\theta_{sl}+f_{la}\cos\theta_{la}\;,$$

where the $\theta_{CB}$ is the static contact angle under the Cassie–Baxter state, $f_{sl}$ and $f_{la}$ represent the surface fractions of the phases of solid–liquid and liquid–air, and $\theta_{sl}$ and $\theta_{la}$ represent the corresponding contact angles. Since the $\theta_{la}$ is 180° in C–B state, so $\cos\theta_{la}=\cos180{}^{\circ}=-1$, then Equation (3) can be rewritten as:

(4) $$\cos\theta_{CB}=f_{sl}\cos\theta_{sl}-f_{la}$$

**Figure 2:**
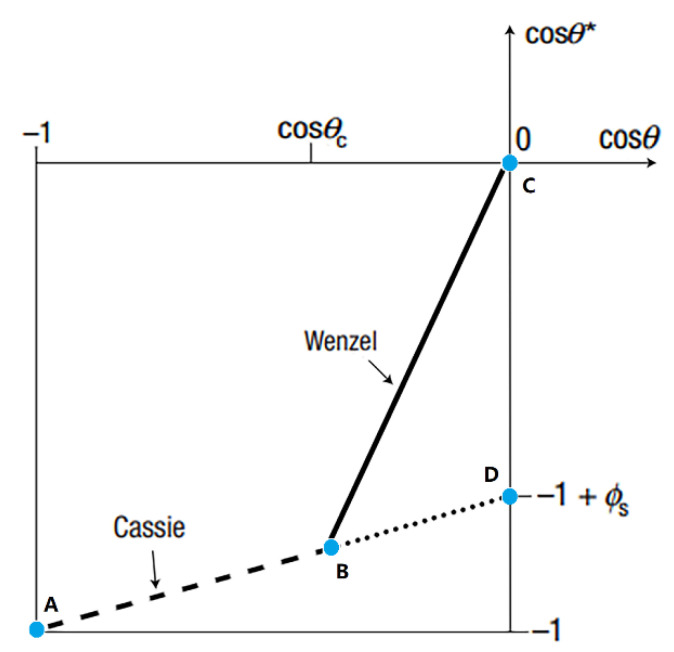
Two models of superhydrophobicity. The solid line and dashed line represent Wenzel state and Cassie–Baxter state, respectively. The dotted line represents the metastable situation where the C–B state can also be observed for θ < $\theta_{c}$. The coordinates of A, B, C, and D are (cos 180°,cos 180°), ($\cos\;\theta_{c},\cos\;\theta^{*}$), (cos 90°,cos 90°), and ($\cos\;90{}^{\circ},\cos\;\theta^{*}$), respectively. Reprinted with permission from Ref. [9]. Copyright@2003, Nature Publishing Group.

Lafuma et al. [9] derived the C–B/W transition in 2003. As shown in Figure 2, where $\theta^{*}$ is the apparent contact angle when minimizing the surface energy of a drop on a rough substrate, θ is the apparent contact angle under Young’s state,$\;\emptyset_{s}$ is the fraction of solid in contact with the liquid, and $\theta_{c}$ is denoted as the critical contact angle between the two states. The coordinates of A, B, C, and D are (cos 180°,cos 180°), ($\cos\;\theta_{c},\cos\;\theta^{*}$), (cos 90°,cos 90°), and ($\cos\;90{}^{\circ},\cos\;\theta^{*}$), respectively. When the apparent contact angle θ is larger than $\theta_{c}$, it follows the C–B state model that the air grooves would be trapped below the drop to form the composite contact. When 90° < θ < $\theta_{c}$, the two states might coexist. Various studies have validated the existence of a metastable state based on simulation and experiment results [15,16,17].

In recent decades, with the development of computer science, computer simulation has become a vital research method. There are numerous methods that can be used to simulate the droplet states on superhydrophobic surface. From the microscale state, molecular dynamics (MD) study the flow behavior of statistical fluid from the perspective of molecular atoms to explore the wetting state transition of droplets at molecular scale [18,19,20,21,22,23,24,25,26,27,28]. The simulation dynamics are sometimes given by the Monte Carlo method. Bryk et al. [29] (2021) found that the effective interface potential method can be used to determine the location of the critical wetting transition. Lopes et al. [30] (2017) presented a Potts model simulation of the C–B/W transition on a surface decorated by a regular distribution of pillars.

Meanwhile, for a larger scopic state, computational fluid dynamics (CFD) are normally widely used. They include the lattice Boltzmann method (LBM) and the finite volume method (FVM). LBM is a good choice from mesoscopic state for the simulation of superhydrophobic surface. There are numerous studies of superhydrophobic surface simulation using LBM, such as contact angle of microdroplets on superhydrophobic surface [31,32,33,34,35,36] and wetting transition between Cassie and Wenzel [37,38]. FVM is a mature algorithm in the field of CFD. It is often used to solve macroscopic state problem instead of wetting state transition.

## 3. Corrections in the Static Contact Angle Model on Heterogeneous Surface

The W and C–B models measure the static contact angle with a solid phase area fraction over the whole surface, but the assumption is not necessarily satisfied for heterogeneous surfaces with complex morphologies [39]. McHale [40] (2007) indicated that the roughness ratio of the W model and frictional contact area are valid only when the surface is isotropic all over with uniform morphology. Therefore, it is very important to build a more accurate and versatile description system to precisely reflect the relationship between the micro-structure criteria and static contact angle. At present, the surface microstructure description system includes a roughness description system, fractal theory description system, re-entrant geometry description system, and contact line description system. Next, we will review the advanced progress in these description systems.

### 3.1. Roughness Description System

**Figure 3:**
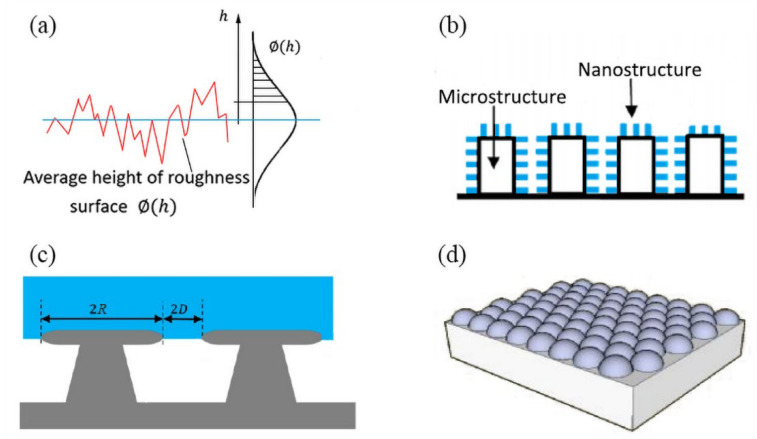
The schematic diagram of microstructures model. (**a**) Roughness model: assuming that the height of rough surface profile obeys Gaussian distribution. Reprinted with permission from Ref. [41]. Copyright@1966, Proc. R. Soc. (**b**) Fractal geometry model. Reprinted with permission from Ref. [45]. Copyright@1967, Science (New York, NY, USA). (**c**) Re-entrant structure model. Reprinted with permission from Ref. [39]. Copyright@2020, Journal of Physical Chemistry. (**d**) Typical nanoparticle coating model. Reprinted with permission from Ref. [46]. Copyright@2002, Nano Lett.

The first statistical description of surface roughness can be traced back to 1966 evolved from tribology. Greenwood and Williamson proposed a theory based on the assumption that the height of the rough surface contour obeys a Gaussian distribution, shown in Figure 3a [41]. However, the detailed textures of a rough surface of a hydrophobic material could elegantly affect its wetting performance. Jiang, et al. [42] (2020) summarized the three typical types of structural morphologies that can change the surface wetting properties: pillar-structured surfaces, pore-structured surfaces, and groove-structured surfaces. Kim et al. [43] (2020) demonstrated that the arrangement of the pattern also had a great correlation with the static contact angle by experiment. They investigated the four shapes of pattern arrangement (triangular, square, hexagonal, and octagonal). Cao et al. [44] (2021) found a correlation between the depth-to-width ratio and static contact angle with the same surface morphology by experiment. As the depth-to-width ratio increases, the air–solid contact area also increases, which leads to an increase in the contact angle.

### 3.2. Fractal Theory Description System

When building a microstructure surface model, dimension scale plays an important role in analyzing the influence of surface microscopic characteristics on the droplet wetting state. There are many scales related to wetting, such as the atomic scale (10^−10^~10^−9^ m), microscopic scale (10^−9^~10^−6^ m), mesoscopic scale (10^−6^~10^−2^m), and macroscopic scale (>10^−2^ m) [47]. However, since the modeling parameters in the statistical model are related to the dimension scale of the rough surface, which is affected by the resolution of the measuring instrument and the sample length of the rough surface, they are important for accurate modeling. Hence, the fractal geometry, which combined the different scales as shown in Figure 3b, is introduced for the analysis of the rough surface contact angle problem [45]. The multi-scaled wetting contact angle of $\theta_{CB}$ and $\theta_{W}$ can be expressed by Equations (5) and (6), based on the fractal theory:

(5) cosθCB=f(Ll)D−2cosθ−fla

(6) cosθW=(Ll)D−2cosθ

where *D* is the Hausdorff dimension, i.e., *D* = log(4)/log(3) = 1.2618, and (Ll)D−2 is the surface area magnification factor [48]. *L* and *l* are the upper and lower limit scales of the fractal structure surface, and θ is the intrinsic contact angle of the material.

Both random and ordered fractal structures have positive influences on the superhydrophobic performance, as the fractal theory illustrated [49,50]. Meanwhile, limited to the self-similarity and self-affinity, the complexity of micro/nanostructures is not satisfactorily described by the fractal theory [45]. Davis et al. [51] (2017) designed three fractal structures, but the results showed no clear correlation between the static contact angle and the fractal dimensions.

### 3.3. Re-Entrant Geometry Description System

Tuteja et al. [52] (2007) demonstrated that there is a third description system related to the wetting performance, called “re-entrant geometry”. It exhibited the capability of supporting repelling behavior to a droplet [53,54]. The re-entrant structures have distinctive features with a wider top and a narrower bottom, shown in Figure 3c. The typical re-entrant geometry can be realized with various possible geometries, such as micro-mushrooms, micro-hoodoo arrays, fiber mats, micro-nail forests, micro-posts, and nanoparticle coatings [39]. The contact angle of micro-hoodoo arrays can be described with the spacing ratio by the following equation:

(7) $$\cos\theta_{CB}=-1+\frac{1}{D^{*}}\left[\sin\theta +\left(\pi -\theta \right)\cos\theta \right]$$

where $D^{*}$ is the spacing ratio, expressed as $D^{*}=\left(R+D\right)/R$, in which *R* is the radius of the micro-hoodoo, D is the half distance between micro-hoodoos. θ is the intrinsic contact angle of the material. For a typical coated nanoparticle model, shown in Figure 3d, the $\theta_{W}$ and $\theta_{CB}$ can be expressed by Equations (8) and (9), respectively [55]:

(8) $$\cos\theta_{w}=\left[1+\frac{\pi }{4\sin\alpha }{\left(\frac{2R}{D}\right)}^{2}\right]\cos\theta$$

(9) $$\cos\theta_{CB}=\frac{\pi }{4\sin\alpha }{\left(\frac{2R}{D}\right)}^{2}-1$$

where *R* is the radius of the nanoparticle. *D* is the distance between the centers of two adjacent nanoparticles. α is the angle of the diamond cell. θ is the intrinsic contact angle of the material.

### 3.4. Fractal Theory Description System

Both the Wenzel and the Cassie–Baxter theories calculate the apparent contact angle from the solid–liquid contact area. The actual measurement results of the contact angle may not be consistent with the two classical theoretical models [56,57]. The solid–liquid contact results from a microscopic surface demonstrated a significant impact of the contact line on the surface wettability. Extrand et al. [58] (2002) proposed that the solid surface energy and the microstructure of the contact line, rather than the inside geometry of the contact area, were the main factors affecting the apparent contact angle. Gao et al. [57] (2007) prepared three groups of microstructures with different morphologies and different surface energies. It was found that the microstructure below the droplet did not affect the contact angle. Oner et al. [59] (2000) found that, when the solid–liquid area fraction was constant, the contact angle would increase as a result of the decreases in the contact length of the three-phase contact line.

## 4. Corrections in the Wetting Transition Mechanism on Heterogeneous Surface

During the last two decades, there has been a drastic upsurge in the research publications related to the correction mechanisms of the C–B/W transition on a heterogeneous surface [60,61,62,63,64]. In this section, the corrections of the wetting transition mechanism are classified from three representative microstructure surfaces: flat-top pillar microstructure, multi-scale microstructure, and re-entrant microstructure.

### 4.1. The Universal Transition Mechanism on Flat-Top Pillar Microstructure

**Figure 4:**
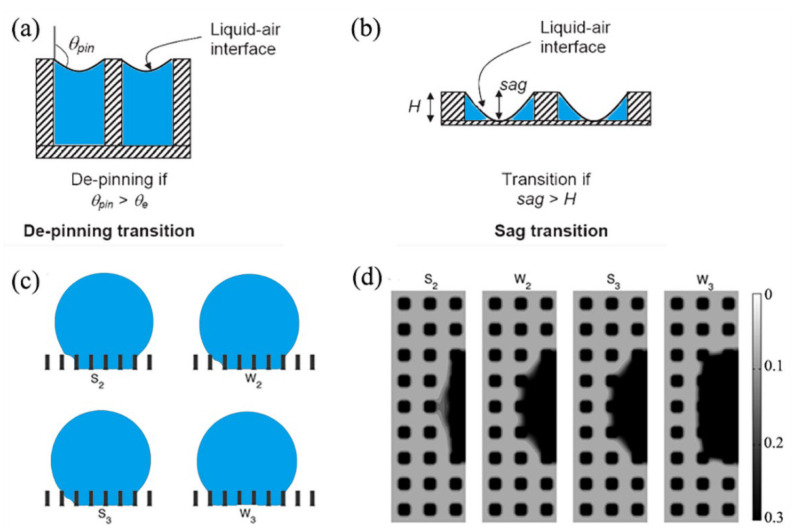
The schematic diagram of the droplet longitudinal propagation in flat-top pillar microstructure for (**a**) depinning transition, and (**b**) sag transition. Reprinted with permission from Ref. [66]. Copyright@2004, Langmuir. (**c**) The schematic diagram of droplet lateral propagation, and (**d**) the simulation results. Reprinted with permission from Ref. [72]. Copyright@2014, Langmuir.

Since the flat-top pillar is the simplest rough structure, it was used as a representative model in the study of wetting transition [65,66]. For the longitudinal propagation of the liquid, there are two ways in which transition can be induced. The first way is a depinning mechanism in which the interface is curved due to the Laplace pressure inside the droplet, shown in Figure 4a. When the hanging interface cannot remain pinned at the pillar edges, the second way of the sag mechanism is induced with the curved liquid–air interface touching the bottom, as shown in Figure 4b [11,65,66,67,68,69,70,71], where $\theta_{e}$ is the intrinsic contact angle of microstructure sidewall, $\theta_{pin}$ is the contact angle of liquid–gas interface pinned at the sidewall of the microstructure, sag is the sag depth at the top of the curved interface, and *H* is the depth of the microstructure. For the lateral propagation of the liquid, Ren et al. [72] (2014) found that the propagation of the liquid front proceeded in a stepwise manner by numerical simulation, shown in Figure 4c,d. Lateral propagation of the liquid front proceeds by one layer of the grooves, from $W_{1}$ to $W_{3}$. $W_{2}$ is an intermediate metastable state (a local minima). $S_{2}$ is the transition state (saddle point) from $W_{1}$ to $W_{2}$, and $S_{3}$ is the transition state from $W_{2}$ to $W_{3}$.

For these flat-top pillar microstructures, various models have been proposed to explain the transition mechanisms and criteria of the C–B/W transition, which can be mainly divided into the thermodynamic analysis and the force-based analysis. Thermodynamic analysis minimizes the Gibbs energy of the system [65,66,73,74], while the force-based analysis establishes the balance of the capillary forces near the three-phase contact line (TPCL) [68,75,76,77].

From the thermodynamic analysis perspective, since the C–B state has a higher energy state than W state, the droplet penetration to the grooves is accompanied by a decrease in the Gibbs energy. This is formed with two components. One is due to the replacement of the solid–air interface with the solid–liquid interface, and the other is due to the change in the liquid–air interfacial area [78,79,80,81]. Although the C–B/W wetting transition is energetically favored, Patankar et al. [65] (2004) conducted a theoretical study to present an energy barrier between the two states on a pillar-patterned surface, which requires extra work to drive the transition with limited kinetics. The barrier energy for the C–B/W transition is given as Equation (10) [65]:

(10) $$G_{B1}=G_{c}-\left(r-1\right)\cos\theta_{e}\sigma_{lv}A_{c}$$

with $G_{c}$ defined in Equation (11):

(11) $$G_{c}=S_{c}\sigma_{lv}-\cos\theta_{r}^{c}A_{c}$$

where $G_{c}$ is the energy of a Cassie droplet on a rough substrate, $G_{B1}$ is the barrier energy for the transition of a Cassie droplet to a Wenzel droplet. $\theta_{e}$ is the equilibrium contact angle of the liquid droplet on the flat surface. $\theta_{r}^{c}$ is the apparent contact angle of a drop under Cassie state. $S_{c}$ is the area of the liquid–vapor contact for a Cassie droplet. $A_{c}$ is the area of contact with the substrate projected on the horizontal plane under the Cassie state. $\sigma_{lv}$ is the liquid–vapor surface energy per unit area. The barrier energy of the C–B/W transition by considering the sag state is given as Equation (12):

(12) $$G_{B2}=G_{w}+\left(1-\emptyset_{s}\right)\left(1+\cos\theta_{e}\right)\sigma_{lv}A_{W}$$

with $G_{w}$ defined in Equation (13):

(13) $$G_{w}=S_{w}\sigma_{lv}-\cos\theta_{r}^{w}A_{w}$$

where $G_{B2}$ is the barrier energy for the Wenzel droplet without forming the liquid–solid contact at the bottom of the valleys. $G_{w}$ is the energy eventually reaching the equilibrium shape of a Wenzel droplet. $\emptyset_{s}$ is the area fraction on the horizontal projected plane of the liquid–solid contact. $S_{w}$ is the liquid–vapor surface area of a Wenzel droplet. $A_{w}$ is the area of contact with the substrate projected on the horizontal plane under the Wenzel state.

From the force-based equilibrium perspective, the resistance to the liquid penetration was considered to be the force produced by the liquid–gas interfacial tension acting on the protrusion side surfaces through TPCL. By considering the force equilibrium between both capillary forces, the influence of gravity and the external pressure, different expressions of the critical pressure were derived to study the C–B/W transition on a micro-structured surface. Xue et al. (2012) gave a theoretical model for predicting the critical pressure on the submersed substrates formed with the cavities and pillars [15]. It was found that both pillars’ and cavities’ geometries existed in the metastable state after depinning. The theory had good agreement with the experiment by Lei et al. (2010), which demonstrated the characteristic size of pillars and that the solid fraction played more important roles than the pillar’s arrangement on the hydrophobicity with higher critical pressure [82]. Zheng et al. (2005) gave the universal critical pressure ($p_{c}$) formulation of pillars as below [75]:

(14) $$p_{c}=-\frac{\gamma f\cos\emptyset_{0}}{\left(1-f\right)\lambda }$$

where λ is the pillar slenderness ratio, f is the fraction of the projection area that is wet, and $\gamma f\cos\emptyset_{0}$ is the water–air interfacial tension.

### 4.2. The Asymmetric Wetting Propagation

Both lateral and longitudinal propagations were investigated for the asymmetric wetting propagation [83,84,85]. For the lateral propagation, Fetzer et al. [86] (2011) conducted experimental work to explain that the lateral asymmetries can be attributed to the curvature of the contact line and the different mechanisms of depinning, such as nucleated jump-like motion and continuous depinning from the sides. Priest et al. [83] (2009) found the asymmetries were attributed to the continuity of the solid component by experiment, and this behavior was consistent with the wettability of chemically heterogeneous surfaces.

**Figure 5:**
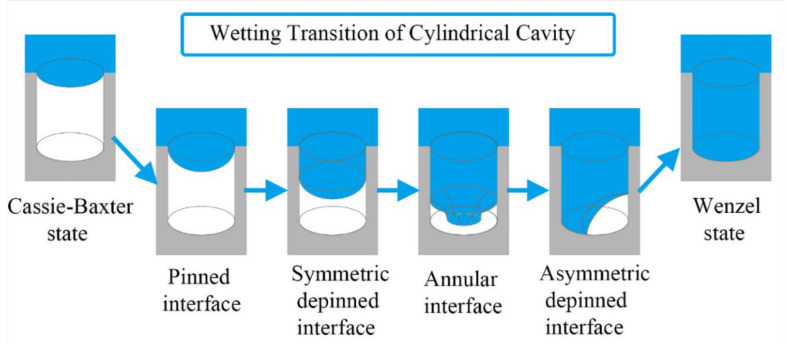
The asymmetric wetting transition mechanism of flat-top pillar microstructure of cylindrical cavity. Reprinted with permission from Ref. [89]. Copyright@2018, the Journal of Physical Chemistry.

For the longitudinal propagation, when the liquid–gas interface touches the bottom of the microstructure, there are two possible contact modes for the sag mechanism: symmetric contact and asymmetric contact [73,85,87,88]. The asymmetric contact shortens the progression of the metastable state to the Wenzel state; hence, it may affect the lifespan of superhydrophobicity [85]. Kim et al. [89] (2018) used a numerical method to find there is an asymmetric depinned stage during the wetting transition process, shown in Figure 5. The wetting transition of a cylindrical cavity begins with an axially symmetrically pinned interface of the liquid and vapor. It is followed by a symmetric depinned interface and then the formation of an annular interface. Finally, the asymmetric depinned interface was formed before reaching the Wenzel state. Giacomello et al. [88] (2012) explained the reason for the asymmetric using the free energy minimization theory. At low filling levels, the interface with the minimized free energy is a straight line, while, for higher liquid volumes in the box, a quarter of the circle occupying one corner offered the minimal free energy.

### 4.3. The Wetting Transition Mechanism on Multi-Scaled Microstructure

Various biomimetic studies found that multi-scaled microstructures can enhance the hydrophobicity of natural surfaces, with a typical example of a lotus leaf [73,90,91,92,93,94,95]. It mainly includes two ways: 1. the droplet infiltrates the nanostructures, 2. multi-scaled microstructure provides more pinning points during the depinning stage. Huang et al. [8] (2013) and Bormashenko et al. [96] (2015) found there is a typical stage that the droplet suspended on the microstructure can infiltrate the nanostructures under lower pressure. Meanwhile, Hemeda et al. [97] (2014) and Xue et al. [15] (2012) discovered that the multi-dimension of microstructures provided more pinning points for the liquid–gas interface during the C–B/W transition. Many studies have investigated these wetting transition mechanisms with different methods. Zhang et al. [98] (2013) and Lee et al. [99] (2016) used the lattice Boltzmann method to investigate the C–B/W wetting transition on the multi-scaled microstructures. Shen et al. [100] (2015) used an experimental method to investigate the wetting transition mechanism on a Ti_6_Al_4_V micro-nanoscale hierarchical structured hydrophobic surface. They demonstrated that the wetting transition process not only increased the apparent contact angle but also decreased the sliding angle significantly. Teisala et al. [101] (2012) used the experimental method to generate a hierarchically rough superhydrophobic TiO_2_ nanoparticle surface by the liquid flame spray. It was found that a wetting transition occurred on a superhydrophobic surface at the nanometer scale.

**Figure 6:**
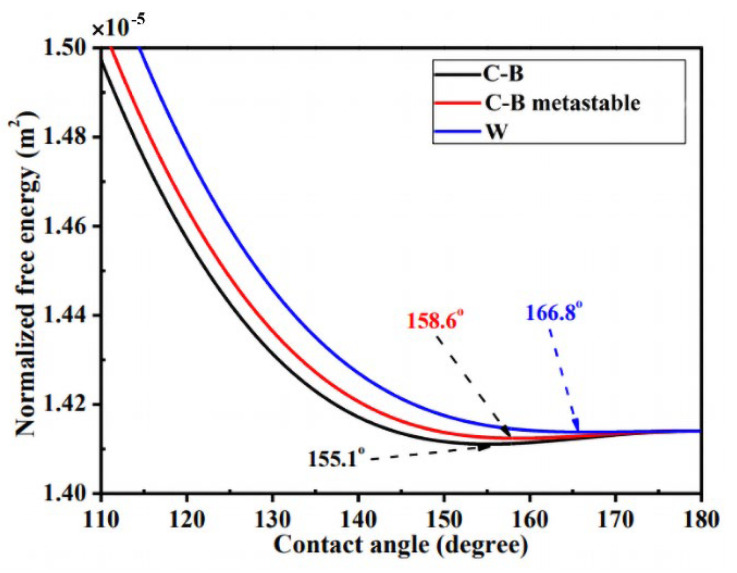
Schematic illustration for the variations in normalized free energy with apparent contact angle for C–B, C–B metastable, and W states on multi-scaled microstructure. Reprinted with permission from Ref. [102]. Copyright@2017, Physicochemical and Engineering.

**Figure 7:**
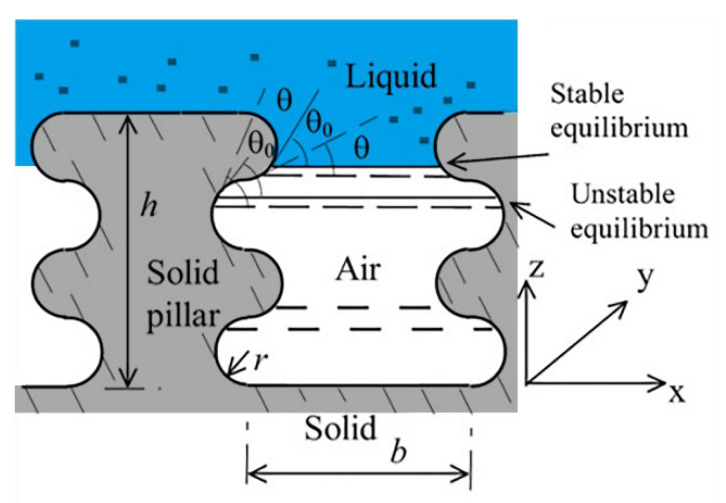
Stability analysis of the composite interface consisting of a rough surface with two-dimensional pillars with semi-circular grooves. Reprinted with permission from Ref. [103]. Copyright@2009, Elsevier Ltd.

The energy models were also used to explain the reason for the transition mechanisms on the multi-scaled surface. Gao et al. [57] (2006) explained that the micro/nanostructure makes C–B state wetting energetically favorable. The additional small-scale roughness on the side surface of the hydrophobic pillars increased the potential barrier for the C–B/W transition, thus making the C–B wetting state more stable. Liang et al. [102] (2017) built a 3-D model to analyze the wetting behavior from a thermodynamics perspective, shown in Figure 6. It shows the variations in normalized free energy (NFE) with apparent contact angle for C–B, C–B metastable, and W states. Here, the NFE decreases at first and then increases with the increase in the contact angle. However, the NFE curve of the C–B state is lower than that of the other two states. Nosonovsky et al. [103] (2009), Hejazi et al. [104] (2013), and Huang et al. [8] (2013) explained this with the capillary mechanisms by both computational and experimental work. They reported that the microstructures or defects on the substrates can significantly increase the wetting hysteresis due to three-phase contact line (TPCL) pinning. As illustrated in Figure 7, the bumps may pin the liquid–air interface because an advance in the liquid–air interface could result in a decrease in the contact angle, which provides the stability of the composite interface.

### 4.4. The Wetting Transition Mechanism on Re-Entrant Microstructure

**Figure 8:**
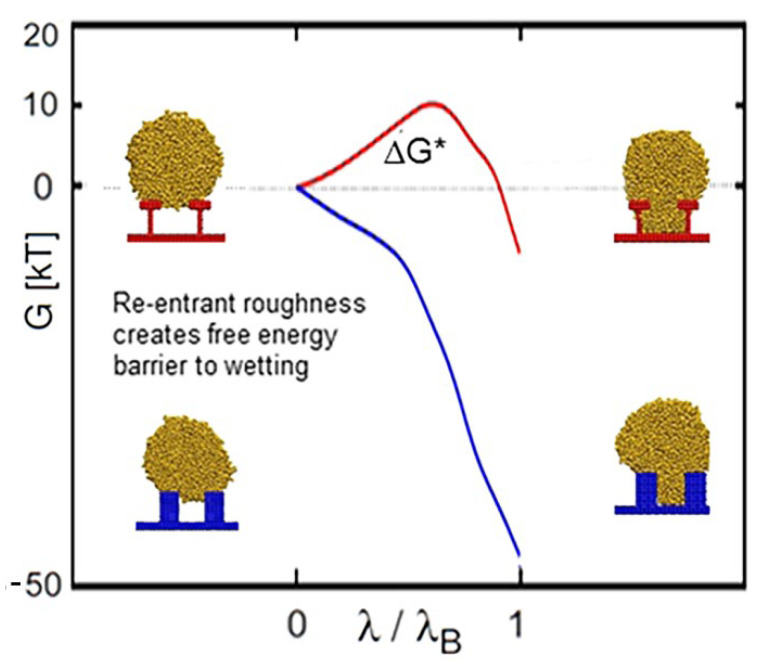
Re-entrant roughness creates a higher energy barrier to wetting. Reprinted with permission from Ref. [108]. Copyright@2012, Langmuir.

Cai et al. [105] (2019) studied three types of nanostructures with different longitudinal-section geometries, including base angles of 60° (inverted trapezoid), 90° (rectangular), and 120° (regular trapezoid). It was shown that the inverted trapezoidal nano-structure surface helped to keep the droplet in the C–B state, in which liquid did not penetrate the nano-structure. This was also described in Refs. [106,107]. Savoy et al. [108] (2012) used a molecular dynamics method to simulate the wetting behavior of different-size droplets on a “T” shape structure via boxed molecular dynamics, which is a technique that is used to quantify the free-energy landscape and estimate the transition rate as the drop moves from one low free-energy basin to another. Further, they found that, at the same height, the “T” structure surface needs to overcome a higher energy barrier than that of the square column surface, which somehow enhanced surface evacuation (shown in Figure 8). Wang et al. [109] (2019) validated the reason for the high superhydrophobicity of a “T” structure by both experimental work and a simulation method. The strong pinning effect on the contact line can significantly change the contact angle and wetting state of droplets.

## 5. Wetting Transition Testing Methods

Lafuma et al. [9] (2003) first proposed a testing method by squeezing droplets with two superhydrophobic surfaces to reflect the wetting stability. This method can be used to observe and study the droplet’s critical pressure and contact angle. The experiment mainly utilizes a micro force sensor on an optical microscope platform. However, this test is only valid for some superhydrophobic materials with poor wetting stability. In recent years, several new versatile strategies were proposed to investigate the wetting transition behavior. The methods include (1) optical, (2) acoustic, (3) confocal laser scanning microscopy, (4) freeze stripping, and (5) high-speed camera methods. The principles and merits of these measurement methods are listed in Table 1, and they will be explained in detail in the following subsections.

### 5.1. Optical Methodology

**Figure 9:**
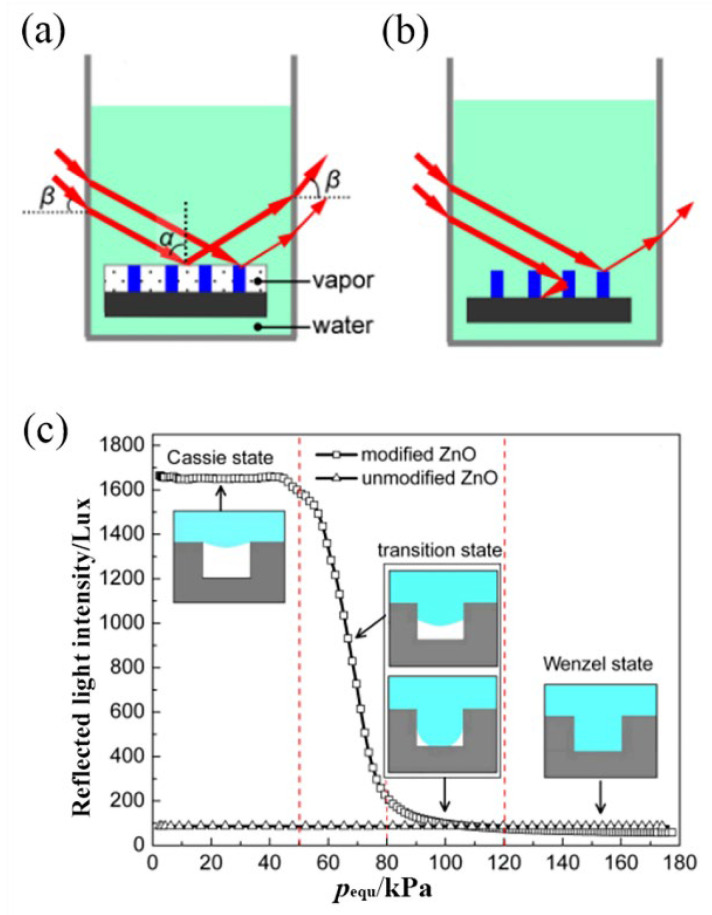
Schematic illustration for the optical reflection methodology: (**a**) the reflection characteristic of Cassie state, (**b**) the reflection characteristic of Wenzel state, and (**c**) the intensity of reflection light vs. equivalent pressure. Reprinted with permission from Ref. [8]. Copyright@2013, Acta Phys. -Chim. Sin.

The optical methodology includes optical reflection and diffraction methodologies. The reflection methodology is the simplest and the most direct way [8,110,111,112,113,114]. It measures the total reflection from an underwater superhydrophobic interface to investigate the wetting behavior and critical pressure of the C–B/W transition. The superhydrophobic interface has a unique reflection property underwater. At the Cassie–Baxter state, the gas–liquid interface satisfies the light total reflection conditions, and its reflected light is relatively bright, shown in Figure 9a. However, at the Wenzel state, since the liquid has penetrated the voids of the microstructure, there is only reflection light at a rough interface, which is darker than that at the Cassie–Baxter state, shown in Figure 9b. Meanwhile, in the meta state, the intensity of the reflected light is between those from the two states. Huang et al. [8] (2013) used reflection methodology to investigate the wetting behavior and to measure the critical pressure of C–B/W transition. Since it is difficult to effectively quantify the intensity of the reflected light by visual observation, laser illumination and photoelectric detection were introduced to quantify the intensity of the reflected light as a function of the reflected light under pressure, as shown in Figure 9c. The critical pressure of the C–B/W transition can be obtained from the inflection point on the reflection curve.

**Figure 10:**
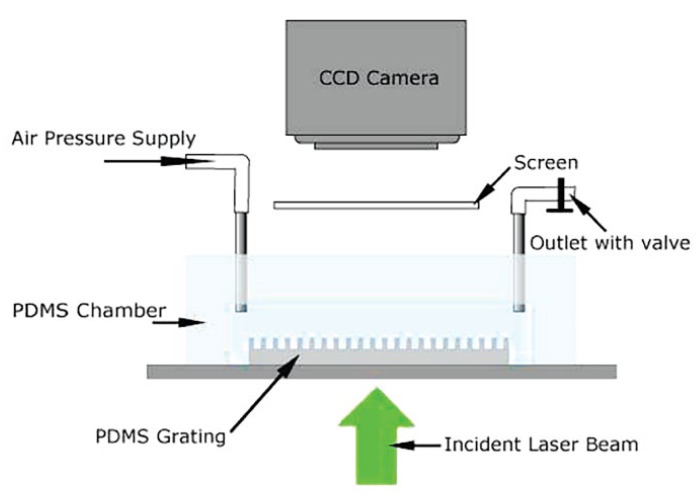
Schematic diagram of the experimental setup for the pressure-dependent observation of diffraction patterns of the water-submerged superhydrophobic grating. Reprinted with permission from Ref. [82]. Copyright@2010, Langmuir.

Compared to the reflection methodology, the optical diffraction methodology can establish the shape of the liquid–gas interface. When a superhydrophobic surface is submerged in water in a fluidic chamber, the surface pattern consisting of regular pillars diffraction can be observed with a laser beam passing through the submerged grating sample in water. The shape of the liquid–gas interface can be indicated by measuring the intensity of several diffraction spots. Lei et al. [82] (2010) employed this method to control and monitor the switching of the C–B/W transition. As shown in Figure 10, water was injected into the chamber with a syringe through the inlet and outlet system. After blocking the outlet valve, hydraulic pressure can be applied through the inlet. A laser beam was aligned to pass through the fluidic chamber and a charge-coupled device (CCD) camera was used to capture diffraction images as a function of the applied pressure. The pressure for the transition between two states can be quantitatively measured.

Through the optical diffraction methodology, Rathgen et al. [115] (2010) studied the microscopic shape, contact angle, and the Laplace law behavior at the liquid–gas interfaces on a superhydrophobic surface.

### 5.2. Acoustic Methodology

**Figure 11:**
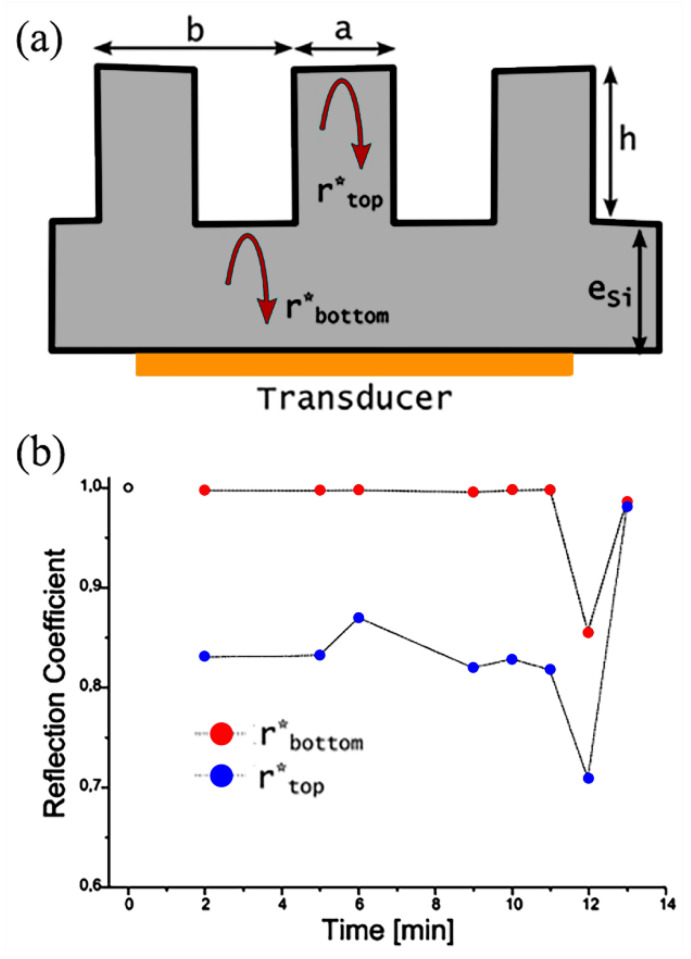
(**a**) Schematic diagram of the experimental setup for the acoustic methodology. (**b**) The evolution of reflection coefficients on the top and bottom interface with the droplet evaporation. Reprinted with permission from Ref. [106]. Copyright@2013, Langmuir.

As the optical methods are neither versatile nor integrable, Dufour et al. [106] (2013) presented an alternative method based on acoustic measurements. An acoustic transducer is integrated on the backside of a superhydrophobic silicon surface with a droplet deposited on the superhydrophobic surface. By analyzing the reflection of longitudinal acoustic waves at the liquid–solid–vapor interface, the transition of C–B/W can be tracked by measuring the reflection coefficient, shown in Figure 11. Here the Pillar dimensions are diameter a = 15 μm, pitch b = 30 μm, and height h = 20 μm, and the thickness of bulk silicon is e_Si_ ≈ 400 μm. The r_top_* and r_bottom_* are the normalized acoustic reflection coefficients at the top and bottom part of a micropillars array, respectively. With a plane acoustic wave propagating onto a micro-structured superhydrophobic surface with two different acoustic media, the absolute value of the reflection coefficient *R*_2/1_ can be calculated from Equation (15):

(15) $$\left|R_{2/1}\right|=\left(\rho_{2}\upsilon_{2}-\rho_{1}\upsilon_{1}\right)/\left(\rho_{2}\upsilon_{2}+\rho_{1}\upsilon_{1}\right)\;$$

where *ρ* is the density of the medium and *υ* is the velocity of the acoustic wave. They also measured the evolution of reflection coefficients on the top and bottom parts of the pillars during the changing of the droplet. The results are shown in Figure 11b. At time = 0 min, an acoustic measurement was performed without a droplet, so the reflection coefficients at the bottom and top interface equal 1. By applying a droplet on the surface at 2 min, the reflection coefficient was reduced to 0.83, while the reflection coefficient of the bottom was not affected. Evaporation occurred after 2 min, and to 11 min. Since the drop was in a meta-Cassie wetting state, no notable change was observed for the reflection coefficients during this time. A spontaneous wetting transition was observed after 11 min with a sudden decrease in the reflection coefficients for both the top and bottom interface. Finally, the drop was evaporated, with the recovery of the coefficients to 1.00. The traces of the coefficients effectively described the wetting transition process. The acoustic methodology was also used to study the wetting transition process with the change in the droplet densities [124].

### 5.3. Confocal Laser Scanning Microscopy Methodology

**Figure 12:**
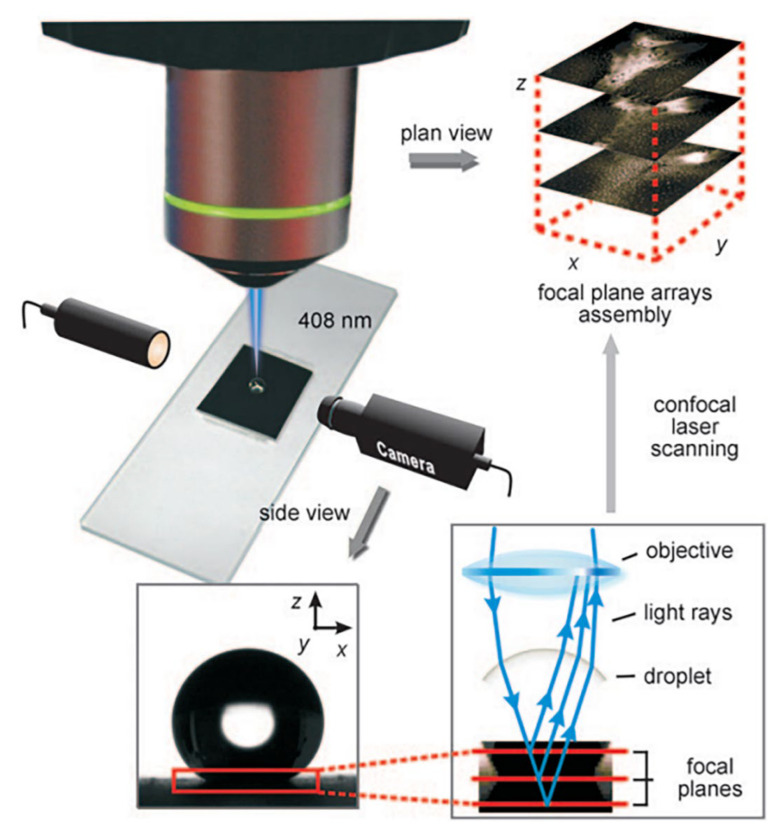
Schematic illustration for the CLSM methodology. Side view and the CLSM 3D plan view for observing a droplet on a superhydrophobic surface sheet. Reprinted with permission from Ref. [116]. Copyright@2010, Wiley-VCH Verlag GmbH & Co. KGaA, Weinheim.

Confocal laser scanning microscopy (CLSM) can continuously record the reflected light during the wetting state transmission through a direct 3D nondestructive imaging method. The setup is shown in Figure 12. The conventional 2D optical observation provides only limited and no semi-quantitative information about the topological complex at the water–gas interface. Since the interface below the liquid cannot be imaged by using a scanning electron microscope (SEM) or transmission electron microscope (TEM), CLSM could provide a better measurement than an SEM, TEM, or atomic force microscope (AFM) [116]. Luo et al. [116] (2010) used a CLSM to observe the air trapped in the buried interface. They presented two approaches to control the wetting state transition by either ultrasonic treatment or introduction of a surface wetting agent, such as sodium dodecylbenzene sulfonate (SDS), into the droplet. Papadopoulos et al. [61] (2013) imaged the dynamic collapse of the C–B state process in detail. They presented the asymmetric contact of the water–gas interface under the metastable evolution process using a CLSM with five detectors.

### 5.4. Freezing Fracture Methodology

**Figure 13:**
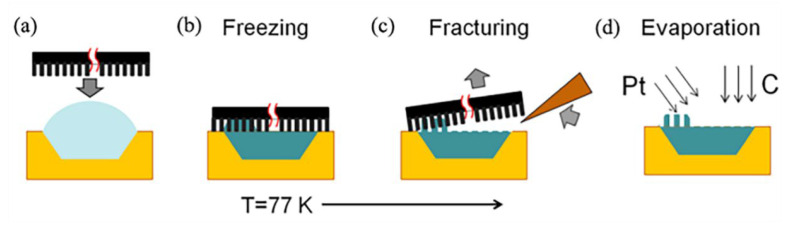
Schematic illustration for the freezing fracture methodology. (**a**) Before freezing, (**b**) rapid freezing of the pure water, (**c**) fracturing, (**d**) surface coated by thin platinum and carbon layers. Reprinted with permission from Ref. [123]. Copyright@2013, Langmuir.

The freezing fracture methodology can be applied for direct observation of the wetting transition process of a droplet on a multi-scaled microstructure [123]. The droplet system at a certain wetting state is immersed in liquid nitrogen, and the droplet would be frozen immediately. The frozen droplet is observed by a scanning electron microscope. The set-up of the experiment is shown in Figure 13. First of all, a liquid droplet of deionized water is deposited into a holder, shown in Figure 13a, a nano-patterned surface of a Si wafer is pressed onto the holder, followed by rapid freezing at 77 K, shown in Figure 13b. Next, the patterned Si wafer is detached from the holder, shown in Figure 13c, which is placed in an evaporation chamber equipped with a cooling stage. Layers of 3 nm Pt and 5 nm C are deposited by electron beam evaporation onto the fracture to avoid sublimation. Ensikat, et al. [127] (2009) applied this method to visualize the contact area between liquids and superhydrophobic biological surfaces. To avoid the sublimation of the droplet and to stabilize the imprint, Cannon et al. [121] (2010) modified the standard method by coating the replica surface with thin platinum and carbon layers.

### 5.5. High-Speed Camera Methodology

**Figure 14:**
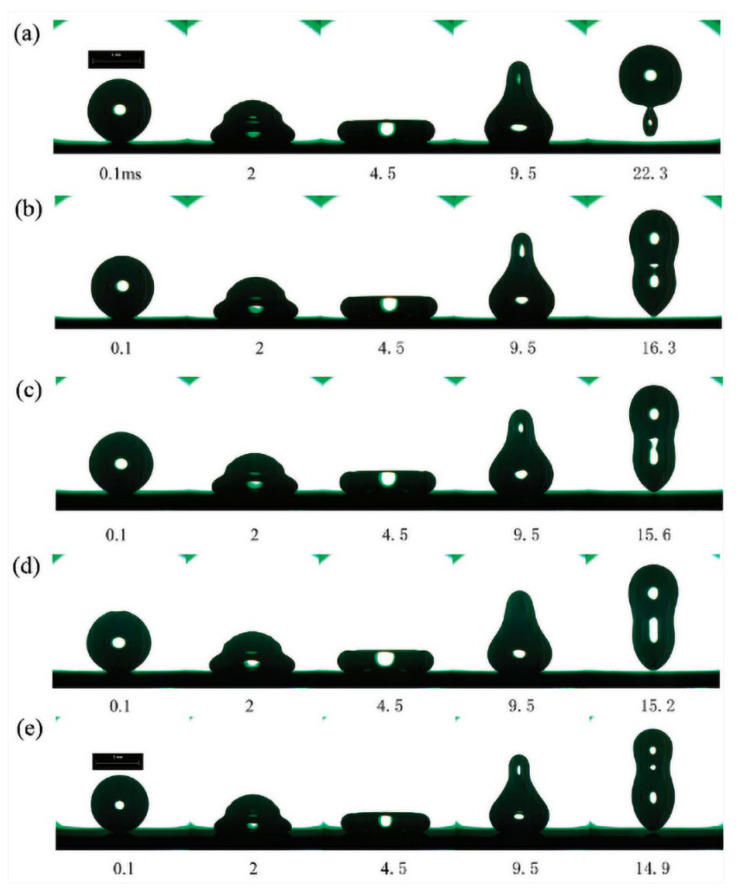
Schematic illustration for the result of high-speed camera methodology. Sequential images show the temporal evolution of the dynamic impact drop on different surfaces. The impact velocity was set up to 0.54 m/s, and then parts (**a**–**e**) represent drop events on the $T_{10}^{20},T_{10}^{40},T_{20}^{40},T_{20}^{80},\;\mathrm{and}\;T_{20}^{100}$ textured surfaces, respectively. Reprinted with permission from Ref. [128]. Copyright@2010, Langmuir.

The high-speed camera can visualize the bouncing of the droplets on a microstructure surface. Hao et al. [120] (2015) investigated the droplet impact dynamics by reflection interference contrast microscopy (RICM) with a wavelength of 546 nm. The process was also recorded by a high-speed camera with a frame rate of 50,000 fps. Li et al. (2010) also used this method to capture the dynamic behavior of the droplet on different surfaces. As shown in Figure 14a–e, there were five textured surfaces $\;T_{10}^{20},T_{10}^{40},T_{20}^{40},T_{20}^{80},\;and\;T_{20}^{100}$, respectively, where the textured surfaces are signified by the $T_{D}^{P}$. *D* is the diameter of the pillars, and P is the distance between to pillars. The image on each patterned surface was very similar except for the counterparts on the $T_{10}^{20}$ and $T_{20}^{40}$ surfaces [128].

## 6. Conclusions and Future Outlook

In this review, a general outline of the fundamental theories on wettability was discussed first, followed by the illustration of recent developments in the static contact angle models for different heterogeneous surfaces. Different description systems were also discussed, including general roughness description, fractal theory description, re-entrant geometry description, and contact line description. Further, the influence of different microstructures on the transition mechanism from the Cassie–Baxter regime to the Wenzel regime has been discussed. The knowledge regarding the available experimental approaches is critical in guiding the selection for different purposes and applications. Therefore, the different measurement approaches are summarized in this review, including optical methodology, acoustic methodology, confocal laser scanning microscopy methodology, freeze stripping methodology, and high-speed camera methodology. A broad outlook for potential future research on the wetting transition mechanism is listed as follows:

- At present, the metastable state is mainly described based on the energy barrier and Laplace pressure, with certain limitations. Most of the theories for the metastable state are based on regular periodic arranged structures. Hence, only the materials with a regular microstructure can have their superhydrophobic properties predicted approximately. Therefore, a thorough understanding of the theory of energy barrier and Laplace pressure on different heterogeneous surfaces is essential.
- There is also an asymmetric contact configuration on the microstructure surface when wetting is metastable. At present, there is a lack of a distinct calculation model for asymmetric instability from a theoretical perspective.
- Most of the classical models on the transition mechanism with fractural geometry and re-entrant geometry assume the droplet perpendicularly impacting the surface. However, inclined surfaces are more common in reality. Hence, future research should focus on droplet dynamics over inclined surfaces.
- The new testing techniques are essential to further discern and identify underlying issues in wetting study since the wetting transition of the superhydrophobic state under pressure is a complicated process. Therefore, future study on advanced testing methods is necessary.

## Figures and Tables

**Table 1:** Summary of wetting transition testing methodology.

Method	Principle	Merits	Reference
Optical reflection	Different wetting states show different light reflection intensity	Simplest and direct	[8,110,111,112,113,114]
Optical diffraction	Change in diffraction pattern reflects the change in gas layer thickness	The shape of the liquid–gas interface can be calculated	[82,115]
Confocal laser scanning microscopy	Scanning the samples by fault section, and three-dimensional reconstruction	Real-time observation of wetting state transition process	[16,61,116,117,118]
High-speed camera	Very short exposure time	High temporal resolution	[11,118,119,120]
Freeze fracture	a certain interface was immersed in liquid nitrogen, and the droplet is frozen rapidly	Small applicable scale for nano-scale microstructure surface	[121,122,123]
Acoustic	The differences of reflection of longitudinal acoustic waves at the composite interface	Versatile and integrable	[106,124,125,126]

## Data Availability

Not applicable.
